# Rapid diagnosis of spinal muscular atrophy using High-Resolution Melting Analysis

**DOI:** 10.1186/1471-2350-10-45

**Published:** 2009-05-29

**Authors:** Wan Jin Chen, Wan Juan Dong, Xiao Zhen Lin, Min Ting Lin, Shen Xing Murong, Zhi Ying Wu, Ning Wang

**Affiliations:** 1 Department of Neurology and Institute of Neurology, First Affiliated Hospital, Fujian Medical University, 20 Chazhong Road, Fuzhou 350005, PR China; 2 Center of Neuroscience, Fujian Medical University, 88 Jiaotong Road, Fuzhou 350004, PR China; 3 Department of Neurology and Institute of Neurology, Huashan Hospital, Shanghai Medical College, Fudan University, 12 Wulumuqi Zhong Road, Shanghai 200040, PR China; 4 Institutes of Brain Science and State Key Laboratory of Medical Neurobiology, Shanghai Medical College, Fudan University, 138 Yixueyuan Road, Shanghai 200040, PR China

## Abstract

**Background:**

Spinal muscular atrophy (SMA) is an autosomal recessive hereditary disorder caused by mutations of the survival motor neuron 1 (*SMN1*) gene. Recently, high-resolution DNA melting analysis (HRMA) with saturation LC Green dyes has become a powerful post-PCR technique for genotyping or mutation scanning. So far, no studies have applied HRMA to the molecular analysis of SMA.

**Methods:**

The exon 7 and the flanking area of the *SMN1 *and *SMN2 *genes of 55 SMA patients and 46 unrelated normal individuals were amplified with asymmetric PCR with unlabeled probe and symmetric PCR without probe, respectively. The saturation LC Green dyes were added to the PCR system. The PCR products were loaded onto the LightScanner system and were melted from 60°C to 95°C slowly. The melting curves were acquired and analyzed by the LightScanner software.

**Results:**

Three types of melting curves that correlated with the presumed genotype of SMA patients and controls were clearly separated on the HRMA chromatogram with the unlabeled probe. The 55 SMA patients and 46 non-SMA controls were identified with HRMA with a 100% clinical sensitivity.

**Conclusion:**

The HRMA with saturation LC Green dyes and unlabeled probe appears to be a suitable, alternative method for the diagnosis of SMA, with high sensitivity and specificity.

## Background

Spinal muscular atrophy (SMA) is a common autosomal recessive hereditary disease characterized by degeneration of the anterior horn α-motor neurons in the spinal cord, leading to paralysis and atrophy of proximal muscles. The survival motor neuron (*SMN*) gene is the disease-causing gene of SMA [1], and it exists as two nearly identical copies, *SMN1 *and *SMN2*. *SMN1 *is the critical gene involved in SMA, as more than 90% of SMA patients have *SMN1 *exon 7 homozygous deletions [1-3]. Homozygous absence of SMN2 genes does not cause SMA and is found in about 5% of normal individuals. In SMA patients, the number of *SMN2 *copies is inversely correlated with disease severity [4-6]. Only five nucleotides differ between *SMN1 *and *SMN2*. *SMN1 *can be distinguished from *SMN2 *by two nucleotide changes in exon 7 and 8, which can be used to detect the deletion of *SMN1 *and establish the diagnosis of SMA. To date, four assays have been described for detecting the absence of *SMN1*: single-stranded conformation polymorphism (SSCP), restriction enzyme digestion analysis, denaturing high-performance liquid chromatography (DHPLC) analysis and liquid microbead arrays. SSCP is a very time-consuming procedure [1,3]. Restriction enzyme digestion analysis is the traditional method for SMA, but its accuracy can be hindered by incomplete digestion [2]. DHPLC is a more recent technology for *SMN1 *mutation screening [7-9], but it is confined to the WAVE Nucleic Acid Fragment Analysis System. While the liquid microbead arrays is a sensitive, high-throughput approach that can be used to detect *SMN1 *exon 7 deletions from blood spots, the cost may prohibit its application in many laboratories [10].

High-resolution DNA melting analysis (HRMA) with saturation LC Green dyes is becoming a powerful post-PCR technique for genotyping and mutation scanning. Apparent advantages of HRMA include speed of analysis and convenience, as there is no need for processing or separation of PCR products. Recent reports indicate that the sensitivity and specificity is 100% for amplicons less than 400 bp, and those for amplicons between 400 to 1000 bp are 96.1% and 99.4%, respectively [11]. In this report, we present the results of a study using the HRMA method to analyze a cohort of SMA patients and controls. To date, there have been no reports using the HRMA method for the molecular analysis of SMA.

## Methods

### Subjects

A total of 55 unrelated patients with SMA and 46 unrelated normal individuals were recruited from April 2005 to April 2008. All of the patients fulfilled the diagnostic criteria [12] and were confirmed to have *SMN1 *exon 7 homozygous deletions by RFLP and DHPLC analysis using the detailed procedure previously described [9,13,14]. Of the 46 normal controls analyzed by RFLP, 3 were homozygous for *SMN2 *exon7 deletions. The remaining 43 normal controls possessed both *SMN1 *and *SMN2*. Furthermore, 12 of the normal individuals were analyzed by real-time fluorescence quantitative PCR, as previously reported [6,15]. Informed consent was obtained from each individual or parents of individuals younger than 18 years. The study was approved by the local ethics commission. Genomic DNA was isolated from peripheral blood lymphocytes using the QIAamp DNA Blood Minikit (QIAGEN, Hilden, Germany).

### Asymmetric and symmetric PCR

The exon 7 and flanking area of the *SMN1 *and *SMN2 *genes were amplified by PCR, the primers were SMNF(5'-AGACTATCAACTTAATTTCTGATCA-3') and SMNR(5'-GATTCACTTTCATAATGCTGG-3'). The length of the PCR product was 241 base pair and contained 2 nucleotide differences between *SMN1 *and *SMN2*. For asymmetric PCR, the 20 μL reaction contained 40 ng of genomic DNA, 200 μM dNTPs, 1.25 U Taq DNA polymerase (Dichuan Biosystem, China), 2.0 ul LCGreen Plus dye (Idaho Technology), 0.05 μM SMN F primer and 0.5 μM excess SMN R primer, and 0.5 μM unlabeled probe to *SMN1*. The sequence of the *SMN1 *probe is 5'-TATAGCTATCTATGTCTATATAGCTAT-P-3', in which the underlined "G" is specific to *SMN1 *and "-P" indicates a 3' phosphate. For symmetric PCR, the reaction was similar to that of asymmetric PCR except that both the SMNF and SMNR primers were 0.5 μM, and the *SMN1 *probe was absent. The PCR reactions were performed in a 9700 Thermal Cycler (Applied Biosystems, Foster City, CA); the conditions included an initial denaturation at 94°C for 2 min, followed by 45 cycles of 94°C for 30 s, 57°C for 30 s, and 72°C for 30 s and a final extension at 72°C for 5 minutes.

### Melting curve acquisition and analysis

The PCR products were transferred to a 96-well plate and put on the LightScanner (Idaho Technology). The samples were first denatured at 95°C and rapidly cooled to 40°C at a rate of 20°C/s, then melted from 60°C to 95°C with a slope of 0.1°C/s. The data was acquired every 1°C for a total of 25 readings. Melting curves were analyzed with LightScanner software (Idaho Technology Inc).

## Results

### HRMA with unlabeled probe

**Figure 1:**
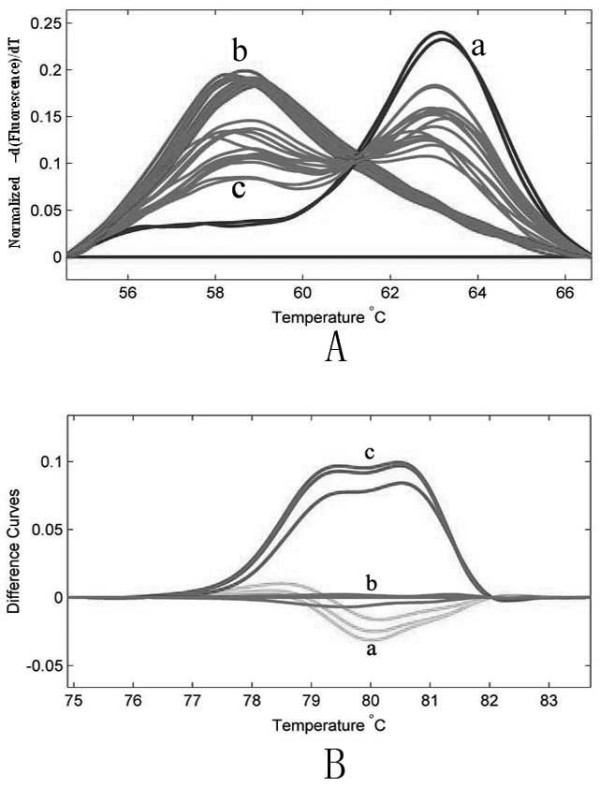
**Results of HRMA with (A) or without (B) unlabeled probe**. a, normal individuals with *SMN2 *deletion; b, SMA patients with *SMN1 *deletion; c, normal controls without *SMN1 *and *SMN2 *deletion (*SMN1/SMN2 *heteroduplexes).

As illustrated in Figure 1A, HRMA displayed three types of melting curve shapes that correlated with the genotype of all the patients and controls. Samples from 55 SMA patients with a confirmed diagnosis of SMN1 exon 7 homozygous deletion had a lower melting temperature due to deletion of *SMN1 *(only possessing *SMN2*), inducing the probe-target mismatch. The three individuals with *SMN2 *deletion (only possessing *SMN1*) had a higher melting temperature, because PCR products were perfectly paired to the unlabeled probe. Melting curves of the control samples with *SMN1 *and *SMN2 *genes showed both duplexes. On the other hand, the results showed no correlation between the height or area of the curves and the copy number of the *SMN1 *or *SMN2 *gene as determined by real-time fluorescence quantitative PCR.

### HRMA without probe

Melting curve profiles of HRMA without probes were available for 6 SMA patients with *SMN1 *deletion, 3 individuals with *SMN2 *deletion, and 6 normal controls without *SMN1 *or *SMN2 *deletion. It was easy to identify the controls with *SMN1 *and *SMN2 *genes according to the melting curve shape, while it was difficult to distinguish patients with a *SMN1 *deletion from normal individuals with a *SMN2 *deletion (Figure 1B).

## Discussion

As already reported, SSCP, restriction enzyme digestion analysis, DHPLC and liquid microbead arrays could be used in detecting the absence of *SMN1*; all of these methods have advantages and disadvantages. Previously, one study showed that HRMA was superior to DHPLC in mutation scanning [16]. In the present study, we performed HRMA with and without an unlabeled probe for the SMA patients and controls. The exon 7 and flanking area of *SMN *gene were amplified. There are 2 nucleotide differences between *SMN1 *and *SMN2 *within this 241 bp product, which correspond to position intron6(-45) (G = SMN1, A = SMN2) and another to exon 7(+6) (C = SMN1, T = SMN2) [17]. According to a previous report [18], the unlabeled probe was designed to target *SMN1*, contained the intron6 (-45) difference in the center and was phosphorylated at the 3' end to prevent polymerase extension during amplification. Asymmetric PCR was applied to improve the rate of hybridization, and the primer ratio of 1:10 referred to in the previous report [18]. The symmetric PCR was performed routinely. Melting transitions were monitored in the presence of LCGreen I, a saturated dye originally designed to detect heteroduplexes [19]. The results showed three types of melting curve that correlate to the presumed genotype (*SMN1, SMN2, SMN1/SMN2*) were clearly separable on the chromatogram of HRMA with an unlabeled probe, and we succeeded in distinguishing the 55 SMA cases from the controls without any error. However, the results of the HRMA without the unlabeled probe were not as good as those with the probe, as it was difficult to distinguish patients with the *SMN1 *deletion from normal individuals with the *SMN2 *deletion.

HRMA with an unlabeled probe has many advantages in detecting the deletion of *SMN1 *compared with SSCP, restriction enzyme digestion analysis, DHPLC and liquid microbead arrays. First, HRMA is sensitive and accurate: the 55 patients with *SMN1 *exon 7 homozygous deletions were detected by HRMA with 100% sensitivity and specificity in accordance with a previous report using this technology [11]. Second, HRMA is easy and rapid to perform, with only four requirements: PCR, saturation LC Green dyes, unlabeled probe and melting instrumentation. Melting analysis can be finished within several minutes.

## Conclusion

HRMA with saturation LC Green dyes and an unlabeled probe seems to be a useful alternative strategy for the diagnosis of SMA. This study has provided "proof of principle" data indicating the utility and sensitivity of HRMA when applied to diagnostic testing for SMA. However, our findings should be replicated in a much larger sampling of SMA patients to assess the specificity and sensitivity.

## Competing interests

The authors declare that they have no competing interests.

## Authors' contributions

WC and WD contributed equally to this work; the contributors are listed in the parentheses: study concept and design (WC, ZW and NW); acquisition of data (WC, WD, X-ZL, ZW, M-TL, SM and NW); analysis and interpretation of data (WC, WD, ZW, X-ZL and NW); drafting of the manuscript (WC, WD and NW); critical revision of the manuscript for important intellectual content (ZW and NW); obtaining of funding (WC, ZW and NW); administrative, technical, or material support (WC, WD, X-ZL, ZW and NW); study supervision (ZW and NW).

## Pre-publication history

The pre-publication history for this paper can be accessed here:



## References

[B1] Lefebvre S, Bürglen L, Reboullet S, Clermont O, Burlet P, Viollet L, Benichou B, Cruaud C, Millasseau P, Zeviani M (1995). Identification and characterization of a spinal muscular atrophy-determining gene. Cell.

[B2] Steege G van der, Grootscholten PM, Vlies P van der, Draaijers TG, Osinga J, Cobben JM, Scheffer H, Buys CH (1995). PCR-based DNA test to confirm the clinical diagnosis of autosomal recessive spinal muscular atrophy. Lancet.

[B3] Wu ZY, Wang N, Mu-Rong SX, Lin MT (1998). Using polymerase chain reaction single strand conformation polymorphism to detect *SMN*^*T *^gene deletions and to confirm clinical diagnosis of spinal muscular atrophy in Chinese. Chin J Neurol.

[B4] McAndrew PE, Parsons DW, Simard LR, Rochette C, Ray PN, Mendell JR, Prior TW, Burghes AH (1997). Identification of proximal spinal muscular atrophy carriers and patients by analysis of *SMNT *and *SMNC *gene copy number. Am J Hum Genet.

[B5] Feldkotter M, Schwarzer V, Wirth R, Wienker TF, Wirth B (2002). Quantitative analysis of *SMN1 *and *SMN2 *based on real-time lightcycler PCR: fast and highly reliable carrier testing and prediction of severity of spinal muscular atrophy. Am J Hum Genet.

[B6] Chen WJ, Wu ZY, Wang N, Lin MT, Murong S-X (2005). Study on the correlation between the *SMN2 *copies and the phenotype of spinal muscular atrophy. Chin J Neurol.

[B7] Sutomo R, Akutsu T, Takeshima Y, Nishio H, Sadewa AH, Harada Y, Mastsuo M (2002). Rapid *SMN1 *deletion test using DHPLC to screen patients with spinal muscular atrophy. Am J Med Genet.

[B8] Mazzei R, Conforti FL, Muglia M, Sprovieri T, Patitucci A, Magariello A, Gabriele AL, Quattrone A (2003). A simple method for diagnosis of autosomal recessive spinal muscular atrophy by denaturing high-performance liquid chromatography. J Child Neurol.

[B9] Chen WJ, Wu ZY, Wang N, Lin MT, Mu-Rong SX (2005). Rapid diagnosis of spinal muscular atrophy using denaturing high-performance liquid chromatography. Zhonghua Yi Xue Yi Chuan Xue Za Zhi.

[B10] Pyatt RE, Mihal DC, Prior TW (2007). Assessment of liquid microbead arrays for the screening of newborns for spinal muscular atrophy. Clin Chem.

[B11] Reed GH, Wittwer CT (2004). Sensitivity and specificity of single-nucleotide polymorphism scanning by high-resolution melting analysis. Clin Chem.

[B12] Munsat TL, Davies KE (1992). International SMA consortium meeting. Neuromuscul Disord.

[B13] Wang N, Wu ZY, Mu-Rong SX, Lin MT (1999). Rapid gene diagnosis of spinal muscular atrophy. Chin J Neuroimmunol & Neurol.

[B14] Chen WJ, Wu ZY, Lin MT, Su JF, Lin Y, Mu-Rong SX, Wang N (2007). Molecular analysis and prenatal prediction of spinal muscular atrophy in Chinese patients by the combination of restriction fragment length polymorphism analysis, denaturing high-performance liquid chromatography, and linkage analysis. Archives of Neurology.

[B15] Chen WJ, Wu ZY, Wang N, Lin MT, Murong S-X (2005). Quantitative studies on *SMN1 *and carrier testing of spinal muscular atrophy. Zhong hua yi xue yi chuan xue za zhi.

[B16] Chou LS, Lyon E, Wittwer CT (2005). A comparison of high-resolution melting analysis with denaturing high-performance liquid chromatography for mutation scanning: cystic fibrosis transmembrane conductance regulator gene as a model. Am J Clin Pathol.

[B17] Lorson CL, Hahnen E, Androphy EJ, Wirth B (1999). A single nucleotide in the SMN gene regulates splicing and is responsible for spinal muscular atrophy. Proc Natl Acad Sci USA.

[B18] Zhou L, Myers AN, Vandersteen JG, Wang L, Wittwer CT (2004). Closed-tube genotyping with unlabeled oligonucleotide probes and a saturating DNA dye. Clin Chem.

[B19] Wittwer CT, Reed GH, Gundry CN, Vandersteen JG, Pryor RJ (2003). High-resolution genotyping by amplicon melting analysis using LCGreen. Clin Chem.

